# Collective predictive coding hypothesis: symbol emergence as decentralized Bayesian inference

**DOI:** 10.3389/frobt.2024.1353870

**Published:** 2024-07-23

**Authors:** Tadahiro Taniguchi

**Affiliations:** ^1^ Research Organization of Science and technology, Ritsumeikan University, Kusatsu, Shiga, Japan; ^2^ Graduate School of Informatics, Kyoto University, Kyoto, Japan

**Keywords:** symbol emergence, emergent communication, predictive coding, probabilistic generative models, Bayesian inference, multi-agent systems, language evolution

## Abstract

Understanding the emergence of symbol systems, especially language, requires the construction of a computational model that reproduces both the developmental learning process in everyday life and the evolutionary dynamics of symbol emergence throughout history. This study introduces the collective predictive coding (CPC) hypothesis, which emphasizes and models the interdependence between forming internal representations through physical interactions with the environment and sharing and utilizing meanings through social semiotic interactions within a symbol emergence system. The total system dynamics is theorized from the perspective of *predictive coding*. The hypothesis draws inspiration from computational studies grounded in probabilistic generative models and language games, including the Metropolis–Hastings naming game. Thus, playing such games among agents in a distributed manner can be interpreted as a decentralized Bayesian inference of representations shared by a multi-agent system. Moreover, this study explores the potential link between the CPC hypothesis and the free-energy principle, positing that symbol emergence adheres to the society-wide free-energy principle. Furthermore, this paper provides a new explanation for why large language models appear to possess knowledge about the world based on experience, even though they have neither sensory organs nor bodies. This paper reviews past approaches to symbol emergence systems, offers a comprehensive survey of related prior studies, and presents a discussion on CPC-based generalizations. Future challenges and potential cross-disciplinary research avenues are highlighted.

## 1 Introduction

**FIGURE 1:**
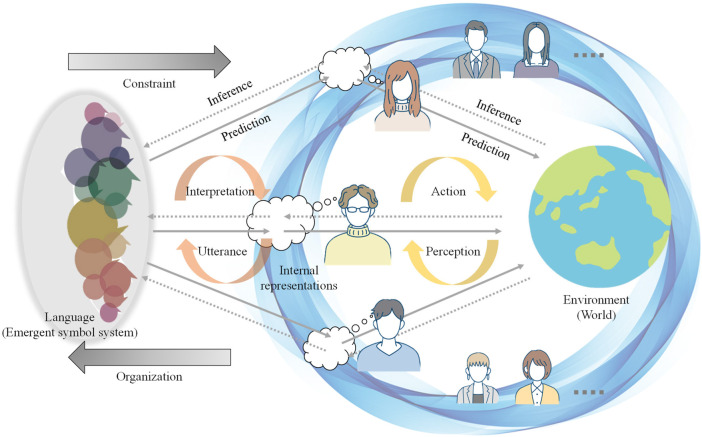
Overview of Collective Predictive Coding. Each agent (human) predicts and encodes environmental information through interactions using sensory-motor systems. Simultaneously, the information obtained in a distributed manner is collectively encoded as a symbolic system (language). When viewing language from the perspective of an agent, each agent plays a role similar to a sensory-motor modality that acts on the environment (world).

Understanding the emergence of symbolic communication is essential not only to unveil the evolutionary origins of language but also to grasp high-level human cognitive capabilities that enable us to communicate and collaborate with others. Humans understand their subjectively experienced world, i.e., Umwelt Von Uexküll (1992), through interactions with the environment based on their sensory-motor system to, subsequently or simultaneously, acquire and use language (Cangelosi and Schlesinger, 2015; Taniguchi et al., 2023a). Thus, while perceiving the world, they form societies through symbolic, especially linguistic, communication^1^. Language is a type of symbol system from the perspective of semiotics, although languages are diverse and complex in terms of syntax, semantics, pragmatics, phonology, and morphology when compared to other types of symbol systems (Chandler, 2002). Through language, humans understand what others perceive and can behave cooperatively as a group. This paper centrally questions why and how humans create languages that dynamically change over time, but function stably in society, realizing communication and collaboration. This study aims to provide a new hypothesis to explain human cognitive and social dynamics pertaining to the creation and upgrade of their shared symbol systems including *language*. Despite the existence of numerous philosophical, psychological, and computational theories, no general computational theory has explained the dynamics of symbol emergence systems. From the evolutionary perspective, explanations must include how the emergence of symbolic communication contributes to the environmental adaptation of humans. Furthermore, the explanation should be consistent with other theories that explain the dynamics of the human cognitive system as a whole. Therefore, this study focuses on those aspects of languages that somehow connect human cognition and promote adaptation to the environment as a multi-agent system. Moreover, by focusing on the emergent characteristics of language, namely, *symbol emergence*, we introduce a new concept of *collective predictive coding* (CPC), through which language development for humans to predict and encode the world in terms of collective intelligence can be studied. Conversely, language itself can be termed as a subject that is coordinated in a distributed manner utilizing human cognitive systems. The situation in which language (symbol system) can be created using CPC is shown in Figure 1. As CPC extends the idea of *predictive coding* (PC) (Hohwy, 2013; Ciria et al., 2021) from individual to society-wide adaptation as a group, we propose the CPC hypothesis. PC posits that the brain predicts sensory information and updates its mental models to enhance predictability. Notably, CPC is shown to be closely related to the *free-energy principle* (FEP), which has gradually gained recognition as a general principle of the human brain and cognition (Friston, 2019; 2010; Clark, 2013), theoretically. The FEP, a broader concept, posits that the brain learns to predict sensory inputs and makes behavioral decisions based on these predictions, aligning with the Bayesian brain idea (Parr et al., 2022). Additionally, the CPC provides a new explanation for why *large language models* (LLMs) appear to possess knowledge about the world based on experience, even though they have neither sensory organs nor bodies.

When considering the emergence of symbol systems that contribute to human environmental adaptation, it is crucial to simultaneously take into account people’s sensory-motor interactions with the environment and their communication through speech and text. The challenge lies in modeling the evolutionary and developmental dynamics of the cognitive and social systems that form the basis for the emergence of symbolic (and linguistic) systems and communications. From both developmental and evolutionary perspectives, knowledge of symbolic (and linguistic) communication does not exist *a priori*. Human infants learn symbolic communication, including language, through interaction with their environment during their developmental stages. Humans, as societies, gradually form symbolic communication systems through evolutionary processes and continuously adjust them in their daily lives. Hence, the quest for symbol emergence or emergent communication should not be limited to the development of a natural language processing (NLP) system (Brown et al., 2020; Kojima et al., 2022; Liu et al., 2023; Min et al., 2023), whose purpose is to create an artificial system capable of processing natural languages appropriately to satisfy certain engineering objectives. Instead, it should be extended to grasp the dynamics of language systems themselves. Highlighting the dynamics and emergence of the semantic aspects of symbol systems, Taniguchi et al. (2016a) proposed the concept of *symbol emergence systems* (SESs) (see Section 2). An SES is a multi-agent system where each agent forms concepts, learns a symbol system such as language, and communicates with other agents. Additionally, a symbol system emerges in a bottom-up manner through communication among agents.

As mentioned above, the phenomenon of symbol emergence involves human linguistic and other high and low-level cognitive capabilities. Thus, the mechanism, i.e., computational explanation, of symbol emergence should be consistent with other principles of cognition, such as PC and FEP, which have gradually gained recognition as general principles of the human brain and cognition (Friston, 2010; Clark, 2013; Hohwy, 2013; Friston, 2019; Ciria et al., 2021). PC is a broadly accepted theory, especially in neuroscience, which has been generalized and is almost synonymous with FEP (Friston, 2019; Friston et al., 2021). Thus, animal brains including that of humans constantly predict sensory information and update their internal representations such as world models, perceptual categories, language models, and motor commands. Clarifying the connection between symbol emergence (or emergent communication) and PC or FEP is crucial, given that symbol emergence is rooted in human cognitive capabilities.

Humans communicate using complex languages that involve numerous characteristics such as syntax, semantics, and pragmatics. Notably, the meaning of a sign can change through long-term interactions with the environment and other agents, depending on the context. The adaptability and *emergent properties* of symbol systems are crucial in human symbolic communication in relation to the principles of *semiotics* as outlined by Peirce (Chandler, 2002). Peirce emphasizes the evolutionary nature of symbols in relation to human development. In this view, the association between a sign and its object is not predetermined but emerges through collective human experience. This perspective also asserts that the categorization of events and objects as members of particular categories is not determined *a priori*. Our human symbolic system is thus characterized by the evolving nature of the relationship between “label” and ”category. Using the terminology of Peircean semiotics, the relationship between signs and objects is fluid and changes according to the interpretant. The exploration of how people form categories and share signs within a community leads us to the framework of SES.

A variety of computational models have been proposed, and numerous studies have been conducted, as described in Section 5, to model the cultural evolution of language and language acquisition in individuals. Such studies follow several types of frameworks, including emergent communication (Lazaridou and Baroni, 2020), multi-agent reinforcement learning (MARL) (Lowe et al., 2017), iterated learning models (Kirby and Hurford, 2002), and symbol emergence in robotics (Taniguchi et al., 2016b). However, a computational model framework that captures the overall dynamics of SES is still necessary. The CPC aims to offer a more integrative perspective, potentially incorporating the pre-existing approaches to symbol emergence and emergent communication.

Another challenge is to understand the potential capabilities of LLMs. Recently, large language models, which are attracting considerable attention in a variety of fields, have not received a satisfactory explanation as to why they are so knowledgeable about our world and can behave appropriately Mahowald et al. (2023). Gurnee and Tegmark (2023) demonstrated that LLMs learn representations of space and time across multiple scales. Kawakita et al. (2023); Loyola et al. (2023) showed that there is considerable correspondence between the human perceptual color space and the feature space found by language models. The capabilities of LLMs have often been discussed from a computational perspective, focusing on the network structure of transformers (Vaswani and Uszkoreit, 2017). However, while the architecture of neural networks can explain the nature of computation, it cannot explain why they possess extensive knowledge about the world as experienced by humans, given their foundation in distributional semantics (Harris, 1954). The knowledge embedded in LLMs arises from distributional semantics, which is an intrinsic part of the language formed by human society. So far, there has been no demonstration or theoretical explanation of how human language has evolved to embed representations of the world within distributional semantics, to the extent that predictive learning in LLMs can effectively decode this knowledge. In other words, how language is formed in the context of human cognition of the world through our bodies, namely, the Umwelt Von Uexküll (1992), and how language systems reflect the structure of the world has not been explained with a viable mathematical model.

To overcome these challenges, we propose the CPC hypothesis, which radically extends the concept of *PC* (Hohwy, 2013; Ciria et al., 2021). This hypothesis expands PC from a single brain to a *group of brains*, suggesting a multi-agent system. It posits that the symbol system emerges as a result of CPC conducted collaboratively by agents in a decentralized manner. In this framework, the emergent symbol system, namely, language, is viewed as a kind of subject, akin to a brain in PC. Within the CPC hypothesis, language is considered a form of collective intelligence, implying that LLMs are directly modeling this collective intelligence. Specifically, the CPC hypothesis argues that symbol systems, especially language, emerge to maximize the predictability of multi-modal sensory-motor information (perceptual experiences) obtained by members of an SES, such as human society. Additionally, the CPC hypothesis regards symbol emergence as a decentralized Bayesian inference, which can be considered as an extension of the Bayesian brain concept to a Bayesian society developed by Doya et al. (2007).

The CPC hypothesis is inspired from the findings of computational studies based on probabilistic generative models and the Metropolis–Hastings (MH) naming game, which is a constructive approach to SESs (Hagiwara et al., 2019; Taniguchi et al., 2023b). The approach provided a Bayesian view of symbol emergence including a theoretical guarantee of convergence. The theory proposed MH naming game as a decentralized Bayesian inference of *external representations* shared among a multi-agent system. This approach is seen as a distinct style of formalizing emergent communication, differing from conventional models that use Lewis-style signaling games, including referential games (see Section 5.1). The former approach is grounded in *generative models*, while the latter relies on discriminative models^2^. However, the broad implications of their approach as a general hypothesis explaining the emergence of symbols in human society were not fully discussed. Therefore, this study establishes a connection between symbol emergence and PC and proposes the CPC hypothesis. The CPC hypothesis posits that self-organization of external representations, i.e., symbol systems, can be conducted in a decentralized manner based on representation learning and semiotic communication ability of individual agents. Additionally, the possible connection between the CPC hypothesis and FEP, stating that symbol emergence follows society-wide FEP, is discussed.

The main contribution of this study is the proposal of the CPC hypothesis, which offers the following features:

1. CPC is a general framework of computational models for SESs based on pre-existing constructive models and their variants. It provides an approach for developing a computational model and introduces a learning algorithm for artificial agents that realize symbol emergence through decentralized communication;
2. CPC hypothesis provides a new computational understanding of symbol emergence in our human society, such as the decentralized Bayesian inference of latent variables shared among agents integrating sensory information of distributed agents and maximizing their predictability;
3. The hypothesis establishes a theoretical connection between PC, FEP, and symbol emergence.
4. CPC provides a new explanation for why LLMs appear to possess knowledge about the world based on experience, even though they have neither sensory organs nor bodies.

The remainder of this paper is organized as follows: Section 2 briefly reviews SESs to provide an integrated view of symbol emergence communication and the representation learning processes of individuals; Section 3 describes the existing probabilistic generative models for symbol emergence; Section 4 describes the CPC hypothesis and its relationship with existing theories; Section 5 briefly discusses other studies that can be considered constructive approaches to SESs; and Section 6 concludes the paper.

## 2 Symbol emergence systems

### 2.1 Overview

Symbol emergence depends not only on social interactions between agents but also on physical (sensorimotor) interactions of individual agents with the environment. For instance, to interpret the meaning of the sign “apple,” an agent must share this sign within its society through social interactions, like semiotic communication, which includes naming the object with others. Concurrently, the agent develops a perceptual category through multi-modal interactions with the object itself. In Peircean semiotics, a symbol is a kind of sign emerging from a triadic relationship between the *sign*, *object*, and *interpretant* (Chandler, 2002). An SES provides a descriptive model for the complete dynamics of symbol emergence (Taniguchi et al., 2016a; Taniguchi et al., 2018 T.) and a systematic of the fundamental dynamics of symbolic communication, regardless of artificial or natural agents.

**FIGURE 2:**
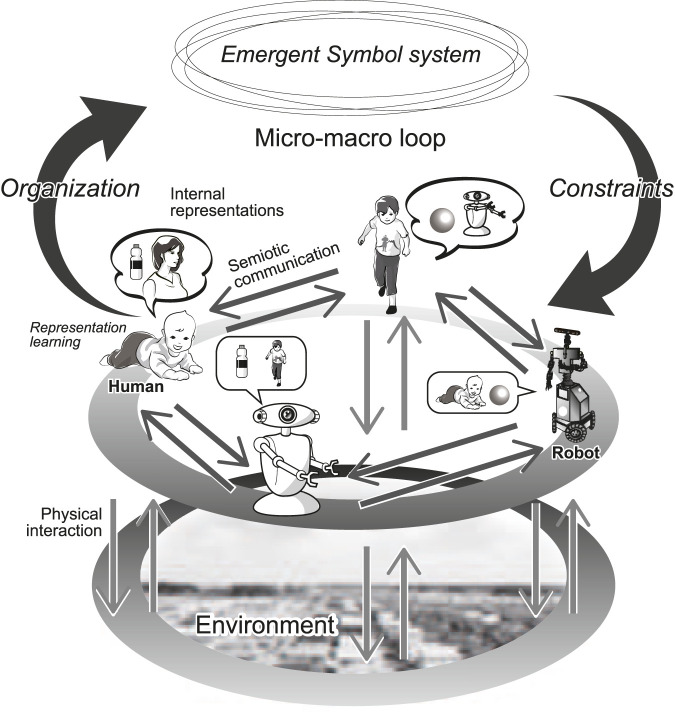
Overview of an SES. Each agent physically interacts with its environment using its sensorimotor system (vertical arrows). Furthermore, every agent has semiotic communication with other agents using signs or symbols (horizontal arrows). Through these interactions, each agent forms internal representations (representation learning). Additionally, an emergent symbol system is organized and shared throughout the system (upward round arrow). To achieve a proper semiotic communication, each agent must follow the rules embedded in the symbol system; communication and perception are constrained by the emergent symbol system (downward round arrow). The total system involves top-down and bottom-up dynamics, often referred to as a micro-macro loop (or effect) in complex systems theory (Kalantari et al., 2020). Further details are discussed in (Taniguchi et al., 2016a).

Figure 2 presents an overview of an SES involving multiple agents that initially consists of a group of humans interacting with their environment through *physical interactions* using their sensorimotor system. They also interact with other agents through semiotic communication using signs. In SESs, interactions based on the exchange of signs between agents are referred to as *semiotic communication*. In this study, symbolic and semiotic communication are considered to be the same. Taniguchi et al. (2016a) proposed the concept of SES to overcome the issues of symbol grounding (Harnad, 1990).

The two types of interactions coincide and affect each other. However, from the perspective of semiotics, *physical interactions* and *semiotic communication* are distinguishable. Generally, the meaning of a sign in semiotic communication depends on its interpretation (i.e., interpretant), and the interpretation heavily depends on a symbol system, which is a cultural existence that people share in the community. Therefore, the information source of a sign’s meaning (i.e., what the sign represents or conveys) depends on and is distributed throughout the symbol system. Conversely, what signals convey in sensorimotor interactions typically does not depend on the culturally shared symbol system within the community^3^. SESs exhibit two main types of dynamics, namely, (*internal*) *representation learning* by individual agents and symbol emergence by multi-agent systems.

In artificial intelligence (AI), except for the studies on emergent communications (see Section 5.1), discussions on language and representation learning have focused primarily on learning by individual agents. Language is not merely a static external linguistic resource that a single agent internalizes and retains. It is distributed throughout society and evolves continuously with use. A symbolic system is neither directly observable nor owned by a single agent; instead, it exists abstractly and is shared across systems in a distributed manner. Humans recognize the world, communicate with others, and are influenced in a top-down manner by language, as illustrated by an arrow labeled “constraints” in Figure 2. While individual cognition, development, learning, and behavior undoubtedly underpin language learning and its use, the language cultivated within society and the dynamics that support it extend beyond individual cognition.

The concept of an SES embodies a model in which a symbol system originates from the environmental adaptations of individuals. However, the system emerges and functions to enable communication among individuals and influence their behavior within the society (Figure 2). As is discussed in Section 2.4, the system possesses *emergent properties* ^4^ in the context of complex systems and is characterized by an internal micro-macro loop (Taniguchi et al., 2016c). It is a distributed autonomous system comprising multiple agents. Notably, the term SES does not represent the symbol system itself but denotes a group of agents with cognitive dynamics that meet certain conditions. Therefore, each agent can be a human or a robot. Moreover, as their cognition is enclosed within sensorimotor systems based on their bodies, they cannot directly observe the internal states of others, nor can they be directly observed or manipulated by external observers. Agents act and continuously adapt to their umwelt (subjective world) (Von Uexküll, 1992).

### 2.2 Learning internal representation based on physical interactions

By integrating multi-modal sensorimotor information to better predict future sensations, people can form *internal representations* ^5^. Even without supervision or linguistic input, humans can learn the physical properties of objects by their autonomous interaction via multi-modal sensorimotor system. For example, without any linguistic input, children can find similarities between different apples because of the similarities in color, shape, weight, hardness, and sounds made when dropped. Such information is obtained through the visual, haptic, and auditory senses. The internal representation learning process or categorization begins before learning linguistic signs, such as words (Quinn et al., 2001; Bornstein and Arterberry, 2010; Junge et al., 2018). Therefore, an internal representation system forms the basis of semiotic communication, and the argument does not exclude the effects of semiotic information provided by another agent during representation learning.

Owing to advancements in neural networks, including deep learning and machine learning techniques in general, progress in computational models for representation learning has successfully produced several types of generative models that directly predict multi-modal sensorimotor information and form distributed internal representations (Suzuki et al., 2016; Pandey and Dukkipati, 2017; Wu and Goodman, 2018). The achieved success and remaining challenges suggest that human cognitive systems form internal representations in a bottom-up manner in interactions with top-down priors (Bengio, 2017; Lake et al., 2017; Taniguchi T. et al., 2018)^6^.

### 2.3 Organizing a symbol system through semiotic communications

Symbols possess inherent arbitrariness in labels. An object labeled as “X” by one agent may not be recognized as “X” by another agent. The crux of symbols (including language) in the human society is that symbolic systems does not pre-exist, but they are developed and transformed over time; thereby forming the premise of the discussion on symbol emergence. Through coordination between agents, the act of labeling an object as “X” becomes shared across the group, gradually permeating the entire society. Symbols exhibit variability in associating signs with objects. An object identified as the sign “X” by one agent may not be recognized as “X” by another. The essence of symbols, including language, in human society lies in the fact that symbolic systems are not pre-existing; rather, they evolve and transform over time, forming the basis for discussions on the emergence of symbols. Through coordination, agents collectively begin to recognize an object as the sign “X,” a concept that gradually becomes widespread throughout society.

Semiotic communication primarily shares the internal states and intentions of agents. However, these internal representations should not be explicitly discretized or directly shared without (arbitrarily designed) signs. Given the flexible nature of symbols, agents negotiate and strive to align symbols. For example, if two agents are jointly attending to a stone and one of them names it “bababa,” if the other agent agrees with this naming, then “bababa” can be agreed to be used as a sign for the object. As similar interactions and agreements proliferate, “bababa” solidifies as a commonly recognized symbol within the multi-agent system. Although this example is the simplest version of negotiation, this kind of dynamics becomes the basis of symbol emergence.

Although a symbol system is a multifaceted system, in its simplest form, it comprises at least a collection of (structured) signs and their associated internal representations of perceptual experiences, events, intentions, abstract concepts, or their relationships. An integral aspect of symbol emergence theory is the inclusion of both syntax and pragmatics as parts of the shared system. However, in this study, our primary focus is on the representational relationships based on the semantic aspect formation of symbol systems. Such an organized structure underscores its emergent nature, with the symbol system developing bottom-up through physical interactions and semiotic communication among agents. This system is then referred to as an ‘emergent symbol system’ (Figure 2), highlighting its emergent characteristics.

### 2.4 Micro-macro loop in SESs

The organization of an emergent symbol system can be considered a self-organization process in the SES. However, the emergence of symbolic systems is not a unilateral, bottom-up process. Instead, it imposes top-down constraints on semiotic communication among agents and on the physical interactions of individual agents, especially on the perception and interpretation of events. Owing to the arbitrariness of symbols, every sensible communication must follow an emergent symbol system involving phonetics, semantics, syntax, and pragmatics shared across the multi-agent system. Agents that do not follow an emergent symbol system cannot benefit from semiotic communication. The effect of a symbol system that emerges in a high-level layer can be regarded as a top-down constraint on a complex system (Kalantari et al., 2020).

Bilateral feedback between higher and lower layers is called the *micro–macro loop* (or effect) (Figure 2). A pattern (or order) in a higher layer is organized in a bottom-up manner through interactions in the lower layer, and the organized pattern imposes top-down constraints on the interactions of the lower layer. This bilateral feedback provides functionality to the system, and the loop is a feature of a complex system with an emergent property used to obtain a function that is not originally discovered by the agents in the lower layer. An *emergent system* is a complex system with emergent properties. Taniguchi et al. argued that symbolic communication emerges as a function of the micro–macro loop in complex systems. Hence, SESs act as the basis for semiotic communication (Taniguchi et al., 2016a; Taniguchi et al., 2018 T.). An SES is a type of emergent system (Kalantari et al., 2020), which is crucial for explaining the emergence of symbolic communication.

An intuitive example can be found in perceptual categorization and naming behaviors. From the perspective of an individual agent, categorization is influenced by the symbol system, such as language, which is learned in a top-down manner. Perceptual categorizations affect semiotic communication, i.e., naming objects, in a bottom-up manner. SESs capture bilateral influence as a part of the total symbol emergence dynamics in a social system^7^

## 3 Probabilistic generative models for symbol emergence

Prior studies aimed at modeling SESs using probabilistic generative models are introduced before proposing the CPC hypothesis. These studies provide a mathematical and computational basis for the CPC hypothesis.

### 3.1 Multi-modal concept formation and representation learning

Constructive computational and robot models exhibiting internal representation learning capabilities are explored. Roy and Pentland (2002) developed a language-learning system based on the multi-modal perception model. Cangelosi et al. (2000) tackled the symbol grounding problem using an artificial cognitive system. Developmental robotics researchers studied language development models (Cangelosi and Schlesinger, 2014). Unlike most classical AI studies in the 2000s and 2010s that mainly focused on a single modality, such as visual, auditory, or linguistic inputs, studies in robotics encompass a wider range of methods as they deal with multi-modal sensorimotor information. Embodied cognitive systems include various sensors and motors, and a robot is an artificial human with a multi-modal perceptual system.

Unsupervised multi-modal categorization and representation learning is an important prerequisite for concept formation and semiotic communication. When considering an “apple,” the concept of an “apple,” is based on multi-modal information. As Barsalou (1999) argued, multi-modal perceptual information is crucial for the basis formation of perceptual categories and learning a grounded language. Accordingly, bottom-up patterns of sensory-motor multi-modal data were associated via the perceptual process, and perceptual symbols, i.e., internal representations, were formed. Recently, it has been discovered that self-supervised learning methods, such as contrastive learning and masked prediction, endow AIs with multi-modal representation learning capabilities without label data Chen and He (2021); Akbari et al. (2021); Radford et al. (2021); Kwon et al. (2022); Nakamura H. et al. (2023). However, even before the emergence of self-supervised learning trends, the field of symbol emergence in robotics had already been exploring multi-modal concept formation and representation learning, using real robots equipped with multi-modal sensorimotor systems. In this context, we revisit these conventional studies.

Researchers studying symbol emergence in robotics aimed to create computational models and robotic systems that performed multi-modal concept formation, including multi-modal categorization through representation learning based on sensorimotor interactions with the environment, such as objects (Taniguchi et al., 2016c; Taniguchi et al., 2018 T.; Friston et al., 2021). Nakamura et al. developed an unsupervised multi-modal latent Dirichlet allocation (MLDA) learning method that enabled a robot to perform perceptual categorization in a bottom-up manner (Nakamura et al., 2009). MLDA is an extension of the latent Dirichlet allocation (LDA), which is a probabilistic generative model widely used in NLP for topic modeling (D.M. Blei and Jordan, 2003), and is a constructive model of the perceptual symbol system. The MLDA system integrates visual, auditory, and haptic information from a robot to form a variety of object categories without human intervention^8^. Thus far, various extensions of the model have been proposed. Nakamura et al. (2011b) proposed a multi-modal hierarchical Dirichlet process (MHDP) that allowed a robot to determine the number of categories. Ando et al. (2013) proposed a hierarchical model that enabled a robot to form object categories with hierarchical structures. The weight of each modality is important for integrating multi-modal information. For example, to form the concept of “yellow,” a color sense is important, whereas haptic and auditory information are not necessary. A combination of MLDA and MHDP methods has been proposed and demonstrated to be capable of searching for appropriate correspondences between categories and modalities (Nakamura et al., 2011a; 2012). Nakamura et al. (2015) also proposed a non-parametric Bayesian extension of these models.Studies on multi-modal categorization provided linguistic information such as utterances to a robot as a type of multi-modal information (i.e., observations of a probabilistic generative model (PGM)). After performing multi-modal categorization, the robot inferred through cross-modal inferences that a word corresponded to information from other modalities, such as visual images. Thus, multi-modal categorization is expected to facilitate grounded language learning (Nakamura et al., 2011b; 2015). Similarly, spatial concept formation models have been proposed by extending the concept of multi-modal object categorization^9^. Recently, owing to the enormous progress in deep generative models, PGMs that exploited the flexibility of deep neural networks achieved multi-modal object category formation from raw sensory information (Suzuki et al., 2016). Thus, high and low-level internal representations (i.e., object and spatial categories and features, respectively) were formed in a bottom-up manner.

**FIGURE 3:**
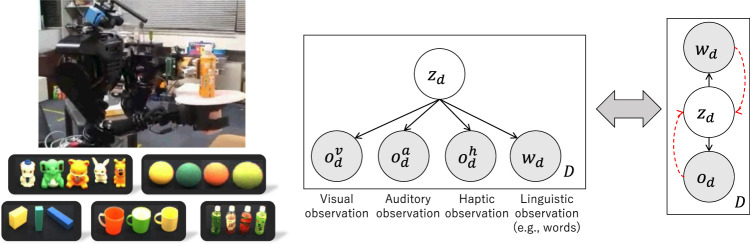
Multi-modal object categorization using MLDA (Nakamura et al., 2009). Left: a robot collecting multi-modal sensory information from objects and samples of formed object categories. Right: Simplified representation of MLDA probabilistic generative model (PGM). Sensory observation for the d th object $o_{d}$ consists of visual $o_{d}^{v}$ , auditory $o_{d}^{a}$ , and haptic $o_{d}^{h}$ observations. Linguistic observation $w_{d}$ , such as words, is also provided as a type of observation. The dotted arrows denote inference processes.

Computational models for multi-modal concept formation in symbol emergence in robotics are based on the mathematical framework of PGM. PGM represents a generative process of observations using multi-modal data and is trained to predict multi-modal information (i.e., model joint distribution). Figure 3 illustrates the PGM of MLDA and an overview of the experiment using a robot (Araki et al., 2012). Latent variables of the PGM were inferred using Bayesian inference. The inference of $p\left(z_{d}|o_{d},w_{d}\right)$ corresponded to the categorization. Thus, the system was trained to predict sensory information and automatically identify categories. Researchers reported that PGM could realize internal representation learning using multi-modal sensorimotor information. Furthermore, the inference of the posterior distribution could be obtained using Markov-chain Monte Carlo (MCMC) algorithms such as Gibbs sampling (Araki et al., 2012). Variational inference was also used to infer the posterior distribution of PGMs for multi-modal concept formation, e.g., Nakamura et al. (2009).

Based on the variational inference perspective, multi-modal categorization (i.e., internal representation learning) and the accompanying optimization criteria are discussed.

$$q^{*}\left(z\right)=\underset{q\left(z\right)}{\mathrm{arg min}}D_{KL}\left[q\left(z\right)\|p\left(z|o,w\right)\right]$$ (1)

where $D_{KL}$ represents the Kullback–Leibler divergence. Instead of the direct minimization of Eq. 1, variational inference is obtained by minimizing the free energy $D_{KL}\left[q\left(z\right)\|p\left(z,o,w\right)\right]$ , suggesting a close theoretical relationship between multi-modal concept formation and FEP.

### 3.2 Symbol emergence through Metropolis–Hastings naming games

To develop a constructive model of the entire SES, the challenge lies in mathematically modeling the process by which a group of agents, while adapting to the environment and engaging in internal representation learning, forms a common symbol system. Conversely, the agents create and share external representations. In this section, we discuss an approach based on PGM, which characterizes the formation of a symbol system in an SES from the perspective of the entire group as it engages in representation learning. Furthermore, this perspective can be interpreted as viewing the emergence of symbols through the lens of *collective intelligence*.

Previous studies on emergent communication employed various types of language games, including referential games, as detailed in Section 5. The naming game is one such language game (Steels, 1995). Hagiwara et al. (2019) offered theoretical insight into naming games and introduced the concept of inter-personal categorization. In their naming game, each agent suggested a name for a target object and communicated the relationship between the names (i.e., signs) and their corresponding classes or attributes. The naming game was inspired from the Metropolis–Hastings (MH) algorithm (Hastings, 1970), a variant of the MCMC algorithm. Subsequently, Taniguchi et al. (2023b) expanded the naming game by dubbing it the MH naming game.

**FIGURE 4:**
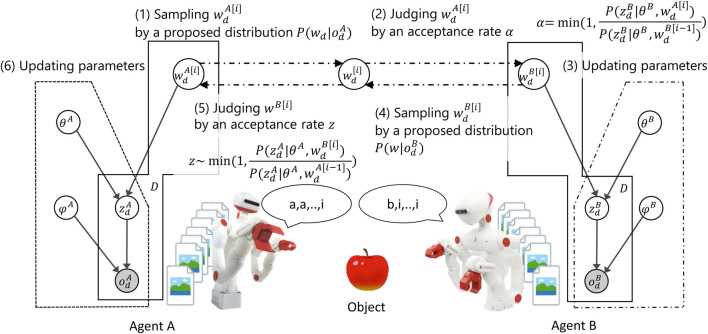
Overview of the computational model of inter-personal categorization proposed by Hagiwara et al. (2019). Top: Integrative probabilistic graphical model for inter-personal categorization. Bottom: Inference of the shared node, $w_{d}$ , is implemented as a naming game based on the Metropolis–Hastings (MH) algorithm. The emergence of symbols is guaranteed to maximize the marginalized likelihood of the sensory observations obtained by the two agents.

In the MH algorithm, a new sample of the latent variable z was drawn as $z^{*}$ from the proposed distribution $q\left(z|z^{\tau }\right)$ at step τ , where $z^{\tau }$ denotes the current state. If the sample $z^{⋆}$ was accepted with probability $A\left(z^{⋆},z^{\left(\tau \right)}\right)=\min\left(1,\frac{p\left(z^{⋆}\right)q\left(z^{\left(\tau \right)}|z^{⋆}\right)}{p\left(z^{\left(\tau \right)}\right)q\left(z^{⋆}|z^{\left(\tau \right)}\right)}\right)$ , the samples converged to the targeted distribution p(z) . The theoretical relationship between the derived naming game and the MCMC algorithm rendered symbol emergence in the game straightforward. The symbol system emerged as the posterior distribution of the latent variable z shared by the agents, conditional on all of their observations. An overview of the MH naming game and its derivation has been discussed, because it forms the basis of the CPC hypothesis. The MH naming game was derived as:

First, the PGM shown in Figure 4 (top) was considered. The PGM represented a hierarchical Bayesian model that integrated multiple types of sensory information. Two types of sensory observation, $o_{d}^{A}$ and $o_{d}^{B}$ , were assumed from the two types of sensors for the d th object. The low-level latent variables $z_{d}^{A}$ and $z_{d}^{B}$ corresponded to $o_{d}^{A}$ and $o_{d}^{B}$ , respectively. The high-level discrete latent variable w integrated two latent variables, $z_{d}^{A}$ and $z_{d}^{B}$ , which represented 2 bits of different sensory information. The total PGM was a simplified version of MLDA (Nakamura et al., 2009), which is a generative model for multi-modal categorization. In the graphical model shown in Figure 4, the two types of sensors were considered two different modalities of multi-modal categorization: auditory and haptic. Assuming that PGM to be a cognitive model of a single agent with two modalities, A and B, the Bayesian inference of $w_{d}$ (e.g., Gibbs sampling, which is a widely used MCMC inference algorithm (Bishop, 2006)) was determined by multi-modal categorization. Thereafter, the internal representations of objects were formed through Bayesian inference to predict multi-modal sensory information.

**FIGURE 5:**
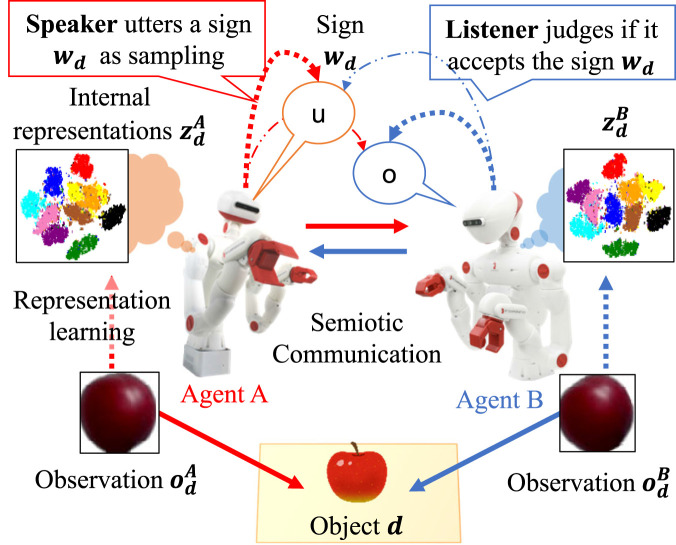
Metropolis–Hastings naming game (Taniguchi et al., 2023b). By presuming joint attention, the naming game, which does not require explicit feedback, operates as a distributed Bayesian inference of latent variables representing shared external representations.

Second, PGM was decomposed into two parts corresponding to sensors A and B, as shown on the bottom of Figure 4. Although the two elemental PGMs shared a latent variable $w_{d}$ , the variables in one elemental PGM were conditionally independent of those in the other. Therefore, when the latent variable $w_{d}$ or its probabilistic distribution was provided, the other variables were inferred independently from another elemental PGM by using Gibbs sampling (Bishop, 2006). Only the shared variable $w_{d}$ was updated depending on the internal parameters of the two PGMs. By exchanging information regarding the probability distribution of $w_{d}$ between two modules, $w_{d}$ was updated. This algorithm represented as an example of the SERKET implementation: a framework for decomposing a large-scale PGM into multiple elemental PGMs (Nakamura et al., 2018; Taniguchi T. et al., 2020). Currently, total PGM is an agent model for multi-modal internal representation learning.

Third, the two PGMs were reconsidered as models of two different agents, where $w_{d}$ represented an external sign. Here, $o_{d}^{X}$ and $z_{d}^{X}$ , with X ∈ {A, B}, were regarded as the observation and internal representations of agent X, respectively. From a generative perspective, PGM indicated $w_{d}$ as a prior information shared by the internal representation of each agent. Thus, metaphorically, language constraints governed the thoughts of each agent in a probabilistic manner and the latent variable was shared among the society members. Inferring the latent variables without referring to the internal variables of another agent remained unclear. When the two elemental modules were considered the cognitive components of an agent, any information found in both PGMs was theoretically available for the inference procedure. Therefore, to update $w_{d}$ , the probabilistic distribution over $w_{d}$ calculated using each module was directly utilized. However, when two elemental modules were regarded as different agents, the internal information of each agent could not be used simultaneously because each was a self-enclosed system, which served as a fundamental constraint in semiotic or symbolic communication.

Surprisingly, an MCMC algorithm for sampling $w_{d}$ without referring to the internal variables of another agent could be derived as a *naming game* (Figure 5). Sample $w_{d}$ , based on the inferred latent variables (i.e., learned parameters) of agent X, was regarded as an utterance of the name of the object agent X interacting with (X, Y) ∈ { (A, B), (B, A) }. The signal was received by another agent Y, who judged whether the name $w_{d}$ was appropriate for the object by considering the latent variables (i.e., internal representations $z_{d}^{Y}$ and learned parameters $\theta^{Y}$ and $\phi^{Y}$ ) of agent Y in a probabilistic manner. If the uttered name was acceptable by agent Y, the internal parameters of Y were updated based on the accepted name $w_{d}$ . Otherwise, the name $w_{d}$ was discarded. When Metropolis choice was adopted as the acceptance ratio, the total naming game was theoretically the same as that of the MH algorithm. Generally, during the naming game, $w_{d}$ , which was highly probable considering the observations and learned knowledge of agent Y, was accepted with high probability. The overall process was a natural computational model for naming games, in terms of language evolution and symbol emergence.

Importantly, from a generative perspective, the total PGM remained an integrative model that combined all the variables of the two different agents. Therefore, the MH naming game worked as an MCMC algorithm (considering strict mathematics), and signs such as words were guaranteed to be sampled from the posterior distribution conditioned on those inputs as the sensory information of the agents, $p\left(w|o^{A},o^{B}\right)$ . Thus, MH naming games could integrate the sensory information of different agents and infer a word as the category of an object similar to that by multi-modal categorization methods used to infer object categories in a bottom-up manner as a Bayesian inference. Further additional algorithmic details are provided by (Hagiwara et al., 2019; Taniguchi et al., 2023b).

Hagiwara et al. (2019) were the first to provide a mathematical basis for bridging symbol emergence involving inter-personal sign sharing and perceptual category formation based on PGMs. The proposed MH naming game guaranteed improved predictability by the SES throughout the multi-agent system (i.e., the SES).

Here, we can consider the possible connection with the free-energy principle. Given that symbol emergence between two agents was considered as decentralized Bayesian inference using MCMC, i.e., the MH naming game, we can possibly consider decentralized Bayesian inference based on variational inference in a similar way. Although a language game that acts as decentralized variational inference has not been invented yet, Ueda et al. (2024) proposed an emergent communication model based on beta-VAE and variational inference. This approach may lead us to a naming game based on variational inference, i.e., decentralized Bayesian inference based on variational inference. From a variational inference perspective, the inference process corresponds to free energy minimization, with the optimization criterion described as follows:

$$q^{*}\left({\left\{z^{k}\right\}}_{k},w\right)=\underset{q\left({\left\{z^{k}\right\}}_{k},w\right)}{\mathrm{arg min}}D_{KL}\left[q\left({\left\{z^{k}\right\}}_{k},w\right)\|p\left({\left\{z^{k}\right\}}_{k},w|{\left\{o^{k}\right\}}_{k}\right)\right]$$ (2)

where variational inference was obtained by minimizing the free energy $D_{KL}\left[q\left({\left\{z^{k}\right\}}_{k},w\right)\|p\left({\left\{z^{k}\right\}}_{k},{\left\{o^{k}\right\}}_{k},w\right)\right]$ , suggesting a close theoretical relationship between multi-modal concept formation and FEP. The aforementioned process was regarded as a PC that uses the total multi-agent system. Thus, symbol emergence corresponded to free-energy minimization throughout the multi-agent system. Although we currently do not have an algorithm for decentralized variational Bayesian inference that can be considered a naming game, I believe suggesting the possible link between the view of symbol emergence based on decentralized Bayesian inference and the free-energy principle is meaningful for future discussions. These findings lead us to propose the CPC hypothesis.

## 4 CPC hypothesis

### 4.1 PC, FEP, and world models

**FIGURE 6:**
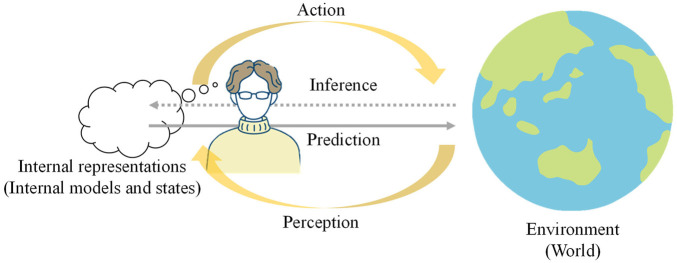
Overview of Predictive Coding. Each agent (human) predicts and encodes the environmental information through interactions using sensorimotor systems. Through the interactions, the agent forms internal models and infers states.

PC is a broadly accepted theory in several disciplines including neuroscience, which posits that the human brain constantly predicts sensory information and updates its mental, world, or internal models to enhance predictability (Hohwy, 2013). During this process, the brain generates predictions of sensory inputs, compares them with actual inputs, and uses the prediction errors to revise the mental model (Figure 6). Furthermore, the aforementioned approach suggests that the brain learns to forecast sensory information and formulates behavioral decisions based on its predictions. A more generalized concept related to such an approach is FEP (Parr et al., 2022), which is associated with the idea of the Bayesian brain proposed by Doya et al. (2007).

From a statistical perspective, prediction errors are naturally interpreted as negative log-likelihoods. For example, least-squares errors are regarded as the negative log-likelihood of normal distributions, ignoring the effects of variance parameters. Hence, minimizing the prediction errors corresponds to maximizing the marginal likelihood, which is a general criterion for training PGMs using the Bayesian approach. In PGMs, latent variables are usually inferred by model joint distributions over observations (i.e., sensory information). Thus, a PGM-based approach usually falls under the umbrella of PC. Considering variational inference, the inference of latent variables z corresponds to the minimization of the free energy $D_{KL}\left[q\left(z\right)\|p\left(z,o\right)\right]$ , where o denotes observations. The FEP proposed by Friston was a generalization of PC (Friston, 2019), which is a general and powerful concept.

The FEP explains animal perception and behavior from the perspective of minimizing free energy. The perceptual state or future actions of animals are defined as latent variables of a cognitive system that continuously interacts with the environment. Free energy emerges when variational inferences of these latent variables are performed. From the perspective of variational inference, the aforementioned PC approximates p(x|o) by minimizing the free energy using an approximate posterior distribution q(x) . This represents the crux of the FEP.

In contrast, world models are representation-learning models that include action outputs (Ha and Schmidhuber, 2018; Friston et al., 2021). An agent is an entity that acts in the world and learns the representation of the world in relation to its actions and understanding of events. Most research on VAEs often considers only sensory information as somewhat static and neglects the temporal dynamics and actions of the agent. World models, rooted in the umwelt of an agent, present the internal representation learning of the agent as it operates within a cognitive world bounded by its sensory-motor information. The relationship between world models and PC or FEP is discussed in detail by Friston et al. (2021); Taniguchi et al. (2023a).

For simplicity, the dynamics in the temporal direction is temporarily ignored (or concatenated into a vector representing a sequence) and the PC of the sensory information is focused. However, arguments that temporal dynamics are essential in contexts such as active inference do exist. Therefore, such extensions were incorporated later by including actions and considering temporal dynamics. For the sensory information o , assuming an internal representation x , the prediction was presented p(o|x) . This recognition was expressed as p(x|o)=p(o|x)p(x) . The former, p(o|x) , represented the generative model, while the latter was referred to as p(x|o) Taniguchi et al. (2023a). The idea behind PC was to recognize the world by predicting sensory information o using the internally predicted internal representation x and to adapt the internal state for predictions.

Notably, thus far, concepts of PC and world models have been utilized primarily to explain and model single-brain cognition and learning capabilities. In contrast, the FEP offers a broader spectrum for explaining the self-organization of cognitive and biological systems (Friston, 2013; Constant et al., 2018; Kirchhoff et al., 2018). However, the relationship between FEP and SES has not been thoroughly described.

### 4.2 CPC

CPC extends the idea of a PC from a single brain to multiple brain regions. In neuroscience, the subject of a PC is a single agent (i.e., the brain of a person). With the emergence of symbolic communication, society has become the subject of PC via symbol emergence. A mental model in the brain corresponds to language (a symbol system) that emerges in society (Figure 1). Decentralized physical interactions and semiotic communications comprise CPC. The sensory–motor information observed by every agent participating in the system is encoded into an emergent symbol system, such as language, which is shared among the agents.

**FIGURE 7:**
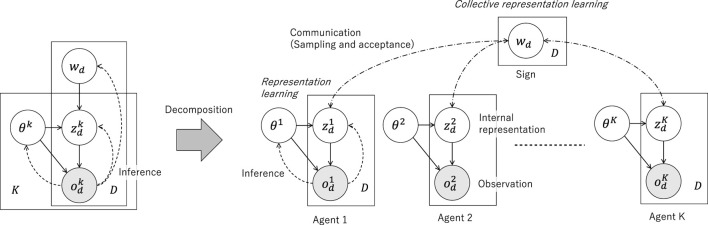
Probabilistic graphical models representing the CPC hypothesis. Left: Probabilistic model that integrates the sensory observations of all agents using a single global variable. The PGM assumes that the observation (i.e., sensory information), $o_{d}^{k}$ , of an agent for the d th object is generated from a word or a sign (e.g., a sentence), $w_{d}$ , *via* an internal representation, $z_{d}^{k}$ , for the k -th agent. The global parameters (including learned parameters representing internal, mental, or world models) of the k -th agent is $\theta^{k}$ . The number of agents and objects are K and D , respectively. Right: Decomposed graphical models. The global variable, $w_{d}$ , is updated via communications, a type of Bayesian inference realization. The MH naming game via inter-personal categorization (Hagiwara et al., 2019) is an example of this approach. Thus, $w_{d}$ is inferred to be a representation of the d th object in a collective manner.

A PGM (Figure 7) was conceptually obtained as an extension of the PGM for interpersonal categorization (Figure 4). Generally, in contrast to representation learning, which is performed by individual agents to form efficient sensory-motor system internal representations for mental model formation, the generative model for symbol emergence frames the process in terms of society (i.e., a multi-agent system) to form a symbol system such as language, representing the sensory-motor information obtained by all agents. In the CPC hypothesis, the emergence of a symbolic system was considered as the *social representation learning*. Moreover, multi-modal representation learning by the brain was mathematically and structurally equivalent to the multi-agent categorization or social representation learning using the SES.

The computational model for CPC was obtained by extending the PGM to interpersonal categorization^10^. Similar to the interpersonal categorization process, we first defined the total PGM by integrating multiple elemental modules. Each elemental PGM was assumed to involve latent variables $z_{d}^{k}$ , and observations $o_{d}^{k}$ corresponding to the k -th agent. If a super system capable of observing the internal variables of every agent existed, a general inference procedure, such as Gibbs sampling or variational inference (Bishop, 2006), could be used to estimate the shared representation $w_{d}$ . However, no such super system exists.

Mathematically, the CPC in the simplest case can be described as follows:

$$\text{Generative model:}\;p\left({\left\{o^{k}\right\}}_{k},{\left\{z^{k}\right\}}_{k},w\right)=p\left(w\right)\underset{k}{\prod }p\left(o^{k}|z^{k}\right)p\left(z^{k}|w\right)\;$$ (3)

$$\text{Inference model:}\;q\left(w,{\left\{z^{k}\right\}}_{k}|{\left\{o^{k}\right\}}_{k}\right)=q\left(w|{\left\{z^{k}\right\}}_{k}\right)\underset{k}{\prod }q\left(z^{k}|o^{k}\right)$$ (4)

Where $q\left(z^{k}|o^{k}\right)$ corresponds to representation learning by the k -th agent, and $q\left(w|{\left\{z^{k}\right\}}_{k}\right)$ is assumed to be estimated through a language game within a group. As a whole, symbol emergence by a group is performed to estimate $q\left(w,{\left\{z^{k}\right\}}_{k}|{\left\{o^{k}\right\}}_{k}\right)$ in a decentralized manner.

Nonetheless, if humans could engage in a language game that allowed inference $w_{d}$ in a decentralized manner, similar to the MH naming game, then a symbol system, such as language, could emerge to integrate the distributed sensorimotor information gathered by individual agents. Thus, a symbolic system arises when agents collaboratively engage in PC.

### 4.3 CPC hypothesis

For the first time, CPC offers a framework that can theoretically and comprehensively capture the entire picture of a symbolic emergence system. By capturing the dynamics of both cognition and society, CPC can holistically explain the dynamics by which language emerges in human society. This depiction provides new hypotheses on the functions imparted by emergent languages. One such hypothesis suggests “Is language/symbol formed to collectively predict our experiences of the world through our sensory-motor system?” The foundation for the dynamics through which language emerges in human society is proposed in this study as the CPC Hypothesis.

Hypothesis 1. **Collective Predictive Coding Hypothesis**

Human language is formed through CPC.

The CPC hypothesis has the following sub-components:

1. *Symbol emergence* is perceived as *social representation learning*, which is a form of *distributed Bayesian inference.* This distributed Bayesian inference is embodied through the autonomous decisions made by each agent to reject or adopt a sign referring to their respective beliefs.
2. Language collectively encodes information about the world as observed by numerous agents through their sensory-motor systems. This implies that distributional semantics encode structural information about the world, and LLMs can acquire world knowledge by modeling large-scale language corpora.

From the perspective of language evolution, the question, “On what cognitive functions did human language evolve?” was analyzed. The CPC hypothesis offers a new explanation for environmental adaptation. Since CPC provides the perspective of environmental adaptation as collective intelligence (Surowiecki, 2004), an extension of PC, more general explanatory principles such as FEP were connected and a one-step extension from PC and the FEP, transitioning from “a system that merely adapts to the environment based on the sensory-motor system” to “a system that forms symbol systems to adapt to the environment based on the sensory-motor system together with others as a society,” was further demanded.

The key concept of the CPC hypothesis is the possibility of decomposing the inference process of an integrative PGM and its structural similarity to the multi-modal concept formation. The decomposed PGMs are shown in Figure 7. The interpersonal categorization model presented an example of a decomposition, wherein the number of agents was K=2 . Hagiwara et al. (2019) suggested a naming game that performed decentralized inference of the shared (emergent) symbol system. Similarly, the process of symbol emergence was regarded as a decentralized inference process involving sharing between multiple agents. Inukai et al. (2023) introduced a recursive MH naming game, which theoretically enabled multiple agents, more than two, to undertake symbol emergence similarly to the MH naming game described by Hagiwara et al. (2019).

The CPC hypothesis has the following implications for the origins of symbolic communication. If humans evolutionarily obtain the capability of internal representation formation, which involves categorization and concept formation, by using multi-modal sensorimotor information and judging the believability of utterances of others based on appropriate probabilities calculated using their own beliefs, they can share signs and optimize the shared external representation system to predict the observations of all agents belonging to the SES. This system is referred to as the emergent symbol system illustrated in Figure 2. Thus, the CPC hypothesis suggests that the ability to determine whether humans believe the statements of other agents based on their own beliefs is crucial for symbol emergence.

At present, we do not have sufficient empirical evidence to support the CPC hypothesis. It is important to design experiments to test the hypothesis in different ways. One approach is experimental semiotics. Okumura et al. (2023) conducted an experiment in which human participants played a naming game similar to the MHNG and showed that the MH acceptance probability predicted human acceptance behavior more accurately than other methods compared. This provides some level of support for the CPC hypothesis. To further test the hypothesis, it is essential to develop computational simulations based on CPC principles and compare the results with human data to validate the model’s predictions.

### 4.4 Society-wide FEP

Considering Figure 7 a deep generative model, CPC was regarded as representation learning involving multi-agent multi-modal observations. Nevertheless, the agents conducted representation learning, in which representations were not only organized inside the brain but also formed as a symbol system at the societal level. Thus, symbol emergence was termed as *social representation learning*.

Since PC could be paraphrased as FEP in many contexts, CPC was viewed from the perspective of FEP. The inference of the latent variables (i.e., representations) was formulated using free-energy minimization considering variational inference. FEP is a general notion of PC and an influential idea in neuroscience as scholars frequently mention that the human brain performs free-energy minimization. Beyond individual FEP, the CPC hypothesis suggests that human society performs free-energy minimization at the societal level by creating symbolic systems. This speculation suggested that symbol emergence was driven by society-wide FEP. Notably, MH naming games based on MCMC algorithm and specific language games that performed variational inference of free-energy minimization have not been invented. However, if decentralized Bayesian inference was viewed from the perspective of variational inference, it would present a society-wide free-energy minimization. This approach clearly provided a theoretical connection between symbol emergence and FEP.

In terms of FEP, the CPC hypothesis suggests that the symbol system emerges by inferring internal representations and shared symbols p(z,w|o) in a decentralized manner considering variational inference. The CPC hypothesis posits that the formation of a symbolic system is the product of collective human intelligence, which is rooted in the capabilities of each agent who allows its engagement in multi-modal representation learning and individually rejects inputs or signs from other agents. Consequently, an emergent symbol system is collectively structured as a system-level (or society-level) representation.

The FEP is not only concerned with the activities of individual brains but is also applicable to collective behaviors and the cooperation of multiple agents. Researchers such as Kaufmann et al. (2021); Levchuk et al. (2019); Maisto et al. (2022) have explored frameworks for realizing collective intelligence and multi-agent collaboration within the context of FEP and active inference. However, the theorization of language emergence based on FEP has not yet been accomplished. Furthermore, CPC represents the first attempt to extend the concepts of PC and FEP by making language itself the subject of PC. Regarding the relationship between language and FEP, Kastel et al. (2022) provides a testable deep active inference formulation of social behavior and accompanying simulations of cumulative culture. However, even this approach does not fully embrace the CPC perspective, where language performs external representation learning utilizing multi-agent sensorimotor systems.

From the viewpoint of FEP, the CPC hypothesis argues that symbol emergence is a phenomenon of free-energy minimization throughout a multi-agent system. In addition, the interpersonal categorization by Hagiwara et al. (2019) suggests the possibility of decentralized minimization of the free energy for symbol emergence. This hypothesis provides direction for future computational studies on symbol emergence, communication, and collaboration between computational studies in language evolution and neuroscience. Additionally, understanding linguistic communication from the viewpoint of CPC enables us to incorporate ideas related to FEP, especially active inference, into language understanding and speech acts, thereby expanding the scope of FEP.

### 4.5 LLM as collective intelligence

Why do LLMs seem to know so much about the “world”? Many studies have suggested that LLMs behave as if they have grounded language (Gurnee and Tegmark, 2023; Kawakita et al., 2023; Loyola et al., 2023) as we briefly described in Section 1. The reason why LLMs are so knowledgeable about our world has not been fully understood (Mahowald et al., 2023). The perspectives offered by the CPC hypothesis give us new speculative thoughts on this question. The CPC hypothesis explains how the language acquired by LLMs is grounded.

In the concept of collective predictive coding, symbol/language emergence is thought to occur through distributed Bayesian inference of latent variables, which are common nodes connecting numerous agents. This Bayesian inference can be performed in a distributed manner without necessarily connecting brains, as exemplified by certain types of language games such as MHNG. Unlike conventional discriminative language games for emergent communication, emergent communication based on generative models (e.g., Taniguchi et al., 2023b; Ueda and Taniguchi, 2024) is consistent with the view of CPC. Thus, even without connected brains, the observations of multiple agents are embedded in a language W . Each utterance $w_{d}\in W$ is sampled from this shared node. Conversely, the prior distribution P(w) over the collection can be considered a language model. As a result, language corpora have distributional semantics (Harris, 1954).

In more computational terms, if representational learning for the i -th agent involves inferring an internal representation $p\left(z^{i}|x^{i}\right)$ for the observation $x^{i}$ , then symbol emergence for a group involves inferring a collective symbol/language (i.e., external representation) $p\left(w|{\left\{x^{i}\right\}}_{i}\right)$ for the observations ${\left\{x^{i}\right\}}_{i}$ . In the context of variational inference, maximizing ELBO with respect to w corresponds to minimizing the free energy for all agent observations ${\left\{x^{i}\right\}}_{i}$ in a group performing symbol emergence, i.e., SES.

Integrating the information in sensorimotor observations of multiple agents’ Umwelts, i.e., self-centered worlds, and forming representations that predict the sensorimotor observations requires more than categorical representations; more complex information representations are needed. Therefore, it is suggested that language adopts compositionality based on syntax. In the conventional work using MHNG, the common node w in Figure 7 has been considered a discrete categorical variable. However, w can be many types of variables, including compositional discrete sequences of variable length, typically found in natural language. In such a case, W becomes a space of (external) representations that model sensorimotor information observed by all agents in the SES.

CPC provides a worldview in which language integrates our internal representations formed through our embodied experiences, corresponding to world models Ha and Schmidhuber (2018); Friston et al. (2021); Taniguchi et al. (2023a), and represents this information as a series or set of sequences. If this is true, then predictive learning of these collections of sequences indirectly models the world experiences we obtain through our human sensorimotor systems. In other words, the latent structure embedded in large-scale language corpora as distributional semantics, which can be learned through language modeling, represents the latent structure of the world. This is because the language system has emerged to represent or predict the world as experienced by distributed human sensorimotor systems. This may explain why LLMs seem to know so much about the ‘world’, where ‘world’ means something like ‘the integration of our environments’.

Recently, LLMs have been considered as candidates for creating artificial general intelligence, and there are also studies focusing on the development of autonomous agents based on LLMs. This has led to a growing perspective that treats LLMs as analogous to individual humans. However, from the viewpoint of CPC, it seems more accurate to consider LLMs as models of *collective intelligence,* which is comprised of the cognitive systems of a large number of people performing PC, rather than as models of an individual cognitive system engaging in PC. Therefore, the CPC hypothesis suggests that LLMs potentially possess the capability to encode more structural information about the world and make inferences based on it than any single human can.

## 5 Related works

This section presents an overview of previous studies on the emergence of symbol systems and language, and examines their relationship with the CPC hypothesis.

### 5.1 Emergent communication

Steels et al. proposed a variety of computational models for language emergence using categorizations based on sensory experiences (Steels, 2015). In their formulation, several types of language games were introduced and experiments using simulation agents and embodied robots were conducted. In a computational experiment conducted in a simulated environment, a group of agents created ways to identify each other using vocabulary-related spatial concepts (Steels, 1995). Steels and Belpaeme (2005) proposed a variety of models to examine mechanisms through which a population of autonomous agents could arrive at a repertoire of perceptually grounded categories. In real-world environments, Steels et al. conducted the “Talking Heads” experiment, where each agent grounded a lexicon to a concept based on visual information to develop a method of communication among agents (Steels, 2015). These experiments showed that language games allowed agents to share lexicons and meanings of simple objects, such as red circles and blue rectangles. Several studies extended the concept of the Talk-Heads experiment. Mobile robots (e.g., AIBO), which have numerous modalities and behavioral capabilities, were used in experiments to learn words and meanings of simple objects and spatial concepts (Steels and Kaplan, 2000; Steels and Loetzsch, 2008). Spranger et al. studied the evolution of grounded spatial languages within a language-game framework (Spranger, 2011; 2015). They implemented a perceptual system for the Sony humanoid robots (Spranger et al., 2012). This study was extended by Vogt et al. from the perspective of semantical and grammatical complexity (Vogt, 2002; 2005; De Beule et al., 2006; Bleys, 2015; Matuszek, 2018). Furthermore, a model for the evolution and induction of compositional structures in a simulation environment was reported (Vogt, 2005).

Since 2016, the advancement of deep learning, particularly its capability for representation learning, has invigorated research on emergent communications based on machine learning Foerster J. N. et al. (2016); Lazaridou et al. (2017a); Lazaridou and Baroni (2020). Trends followed until 2020 were discussed in detail by Lazaridou and Baroni (2020). The group led by Lazaridou and Baroni achieved significant results. Constructive models, such as signaling and reference games, were frequently employed to encourage the emergence of language Lazaridou et al. (2017b); Havrylov and Titov (2017). Primary research focused on the formation of languages with compositional structures were conducted in which simple representations (such as words) were combined to form complex sentences.

In the same way as the CPC extends the idea of generative model-based emergent communication in the joint-attention naming game (Okumura et al., 2023) to population level, models of emergent communication based on Lewis-style signaling games have been extended to populations (Chaabouni et al., 2021). It has been revealed in populated signaling games that larger communities tend to develop more systematic and structured languages (Michel et al., 2022). Moreover, Rita et al. (2021) introduced the idea of partitioning, which separates agents into sender-receiver pairs and limits co-adaptation across pairs, demonstrating that such structure leads to the emergence of more compositional language.

One issue involved the reliance on reinforcement learning (RL)-like feedback principles based on “success/failure of communication.” In Shannon’s information theory, understanding the symbol system was confined to forming a communication channel Shannon (1948), either between two parties or among multiple parties. However, their perspective did not adequately capture the emerging characteristics of symbols in social systems. The communication model foundation relied heavily on the Shannon–Weaver type, where the success or failure of communication served as feedback, rewriting the codebook (relationship between the sign and object) of the speaker or listener. Such a view of language acquisition was criticized by researchers such as Tomasello, who stated that the approach was not a valid metaphor for explaining child language development Tomasello (2005). Before experiencing vocabulary explosion, human infants engage in joint attention. Csibra and Gergely (2009) highlighted that children pre-suppose the intention that parents are trying to teach them when integrating instructions from parents into their learning. Rather than being post-communicative as in reference games, shared attention and teaching intentions were foundational in language development.

From a computational perspective, most studies of emergent communication employed *discriminative models* to represent semiotic communication. The receiver was required to distinguish between the targeted objects. Contrarily, the CPC hypothesis is based on *generative models*. The objective of symbol emergence was not merely the “success of communication,” but rather “organizing a symbol system to better predict or understand the world.” This distinction was significant from a philosophical perspective.

### 5.2 Multi-agent reinforcement learning

The MARL framework was used to model the emergence of symbolic communication. In MARL-based studies of symbolic emergence communication, agents were allowed to output signals as a particular type of action, whereas other agents were allowed to use them as additional sensory information. Such an approach had a long history of application. However, after the introduction of deep RL, RL systems could easily use emergent signals to solve RL problems, benefiting from representation learning (i.e., feature extraction), which is a capability of neural networks.

From the perspective of environmental adaptation, communication fundamentally alters the behavior of others using signs emitted by oneself and changes one’s own behavior based on signs received from others, thereby realizing adaptive behaviors for the group as a whole. This concept was modeled using MARL, which included emergent communication. With recent advancements in deep RL, flexibly interpreting the meaning of signals issued in communication using the representational capability of deep learning has been possible Buşoniu et al. (2010).

Research on symbol emergence using deep-learning-based MARL, such as differentiable inter-agent learning (DIAL) (Foerster J. et al., 2016) and CommNet (Sukhbaatar et al., 2016), has gained momentum since the mid-2010s. Several methods have been proposed, including multi-agent deep deterministic policy gradient (MADDPG), an extension of the deep reinforcement learning method known as deep deterministic policy gradient (DDPG) (Lillicrap et al., 2015; Lowe et al., 2017). These studies were focused on the formation of efficient communication channels for collaboration (Jiang and Lu, 2018; Kilinc and Montana, 2018; Iqbal and Sha, 2019; Kim et al., 2019; Kim et al., 2021). Often, the success of communication in a given MARL task is evaluated by the achieved performance, specifically the amount of reward obtained, with less attention paid to the structure of the emergent language.

When viewed as a model of language emergence, research on symbol emergence based on multi-agent reinforcement learning produced languages that were task dependent. Thus, such issues require attention when considering language emergence.

A theoretical connection exists between the MARL and PGM-based approaches (i.e., PC). Within the past decade, the concept of control as a probabilistic inference (CaI) has gained significant attention (Levine, 2018). CaI allowed for the reformulation of RL as a probabilistic modeling of Bayesian inference. Considering the reward function as an energy function of the probability distribution of optimality ${\bar{o}}_{t}$ : $p\left({\bar{o}}_{t}=1|s_{t},a_{t}\right)=\exp\left(r\left(s_{t},a_{t}\right)\right)$ , made optimal action selection (i.e., policy search) equivalent to the Bayesian inference of future actions: $p\left(a_{t}|s_{t},{\bar{o}}_{1:T}\right)$ . Several model-based RL methods have been interpreted as variational inferences of a future trajectory $p\left(\tau |{\bar{o}}_{1:T}\right)$ , where $\tau =\left(s_{t+1,T},a_{t,T}\right)$ (Okada and Taniguchi, 2020).

Here, we considered partially observable Markov decision process (POMDP) settings, which are more general settings in RL where the state $s_{t}$ is estimated from observations $o_{1:t}$ . In RL, we were interested in a latent state sequence $s=s_{1:T}$ , a future action sequence $a=a_{t,T}$ , and observations up to the present $o=o_{1:t}$ , assuming a sequence of optimalities $\bar{o}={\bar{o}}_{1:T}$ . Thus, we demonstrated that RL could be considered as a PC and Bayesian inference of latent variables, that is, states and a future action sequence, respectively. By introducing CaI into MARL, emergent communication was formulated in terms of PGM-based modeling (i.e., the context of PC performed by multi-agent systems).

Thus, the aforementioned interpretation paved the way for extending MARL from a CPC viewpoint. For simplicity, we included optimality $\bar{o}$ as part of the observations o . Emergent communication in RL was suggested to be interpreted as a probabilistic inference of the shared variable w , that is, $p\left(w|{\left\{o^{k}\right\}}_{k}\right)$ . From this perspective, emitting a message $w_{t}^{k}$ corresponded to sampling a message from the posterior distribution $p\left(w|{\left\{o^{k}\right\}}_{k}\right)$ . From the viewpoint of variational inference, emergent communication (i.e., symbol emergence in MARL) estimated the approximate distribution q(w) for the prior distribution $p\left(w|{\left\{o^{k}\right\}}_{k}\right)$ as

$$\left.q^{*}\left({\left\{z^{k}\right\}}_{k},w\right)=\underset{q\left({\left\{z^{k}\right\}}_{k},w\right)}{\mathrm{arg min}}D_{KL}\left[q\left({\left\{z^{k}\right\}}_{k},w\right)\|p\left({\left\{z^{k}\right\}}_{k},w|{\left\{o^{k}\right\}}_{k}\right)\right)\right]$$ (5)

Optimization was performed by minimizing the free energy $D_{KL}\left[q\left(z,w\right)\|p\left(z,w,o^{\prime }\right)\right]$ . Thus, a multi-agent system-wide free-energy minimization was interpreted. For example, Nakamura T. et al. (2023) and Ebara et al. (2023) extended the MH naming game and proposed a probabilistic emergent communication model for MARL.

### 5.3 Iterated learning models

The iterated learning model (ILM) emulates the process of language inheritance across generations and seeks to explain how compositionality in human languages emerges through cultural evolution (Kirby, 2001). The ILM has been validated using agent-based simulations (Kirby, 2001; 2002; Kirby et al., 2015), mathematical models (Brighton, 2002; Griffiths and Kalish, 2007; Kirby et al., 2007), and laboratory-based language evolution experiments (Kirby et al., 2008; Scott-Phillips and Kirby, 2010; Kirby et al., 2015). ILM models the social transmission of knowledge from parent to child generations. Specifically, the process through which the language and cultural knowledge of one generation is passed on to the next is modeled, allowing a compositional study on how language and culture evolve over time. While research on emergent communication, multi-agent RL, and symbol emergence robotics has often focused on the learning capabilities of individual agents, ILM adopts a more holistic view by examining the transmission of language and culture through society. Thus, an approach akin to complex system simulation research is offered, providing a compositional understanding by observing phenomena that arise through interactions among groups of agents. The theoretical significance of ILM suggests that the unique compositional language of humans can be reduced to a learning ability that does not pre-suppose linguistic compositionality but is based on specific linguistic functions. However, ILM does not address how the resulting languages represent and segment the world, especially in terms of continuous, multi-modal perceptual information such as images and sounds, and how they contribute to the environmental adaptation of agents.

Unlike CPC, ILM does not concentrate on representation learning but places more emphasis on the social transition of linguistic knowledge between generations. Incorporating intergenerational communication into CPC is a direction for future research. Theoretically, the integration is feasible. If realized, CPC can be emphasized as a comprehensive framework that captures the dynamics modeled by the ILM. However, forging a concrete connection between the CPC and ILM remains a challenge.

### 5.4 Symbol emergence in robotics

Symbol emergence in robotics is a constructive approach for *SESs* (Taniguchi et al., 2016c). Central to these discussions is the question of how robots equipped with sensory-motor systems (embodiment) segment (differentiate) the world, form concepts based on subjective experiences, acquire language, and realize symbol emergence.

As introduced in Section 3, models have been proposed for the formation of orients/concepts based on multi-modal information. Such methods focused on the formation of internal representations based on multi-modal information. An environment even without a pre-existing symbol system can exist. Therefore, numerous studies have integrated multi-modal information such as visual, auditory, and tactile data to form the concepts of objects and locations (Nakamura et al., 2009; 2011a; 2015; Taniguchi et al., 2017b; Taniguchi et al., 2020 A.).

Symbolic communication involves exchanging arbitrary signs. SESs require agents to segment continuous vocal sounds into words as clusters of arbitrary symbols for language acquisition. Furthermore, research on automatically discovering word units and acquiring vocabulary by obtaining unsegmented sound sequences, together with multi-modal information related to objects and places, has been conducted (Nakamura et al., 2014; Taniguchi A. et al., 2018). Although word units can be discovered from character strings using unsupervised learning (Goldwater et al., 2009; Mochihashi et al., 2009), such approaches have been extended to consider speech input as an observation. PGMs and inference methods have been proposed to analyze the two-layer structure (dual segmentation structure) unique to a language, consisting of phonemes and words, and simultaneously estimate phonemes and words through unsupervised learning (Taniguchi et al., 2015; 2016d).

Existing studies demonstrated that PGM-based approach could achieve word discovery and lexical acquisition from continuous perceptual sensory information. Thus, the concept of PC could explain the learning process of signs. However, such studies did not consider the emergence of signs (i.e., bottom-up formation). Each robot learned phonemes and words assuming that the system of signs was fixed. Hence, the lists and distributional properties of phonemes and words were fixed. Therefore, these studies were insufficient for modeling the emergence of symbolic communication.

However, discussions on symbol emergence in robotics that evolved throughout the 2010s primarily focused on multi-modal concept formation and language acquisition by individual robots. They were unable to address the emergence of symbols (languages) in society. Following the discussion in Sections 3 and 4, CPC could be extended to the frontiers of symbol emergence in robotics (Taniguchi, 2024).

## 6 Conclusion and discussion

This study proposes the CPC hypothesis. First, the SES was revisited, providing an integrative view that encompasses both individual internal representation learning and the emergence of symbols, i.e., external representations, for communication. This serves as preliminary knowledge to clarify the complete phenomenon of symbol emergence. Second, multi-modal concept formation based on (probabilistic) generative models was revisited, and a generative emergent communication model, symbol emergence through MHNG, was explained as an extension of internal representation learning, called interpersonal categorization. Third, by extending the idea of interpersonal categorization, we propose the CPC hypothesis, which posits that symbol emergence in a multi-agent system can be regarded as decentralized Bayesian inference through language games. This can be considered social representation learning, as well. This is computationally analogous to the representation learning of multi-modal sensory information conducted by an individual agent, with social representation learning performed through CPC in the same manner as by individual PC. The connection to FEP and LLMs was also discussed. Fourth, four branches of research related to computational models for symbol emergence are introduced: multi-modal categorization and representation learning, word discovery and lexical acquisition, language-game-based approaches, and MARL-based approaches.

The advantage of the CPC hypothesis is its generality in integrating preexisting studies related to symbol emergence into a single principle, as described in Section 5. In addition, the CPC hypothesis provides a theoretical connection between the theories of human cognition and neuroscience in terms of PC and FEP.

The limitations of the CPC hypothesis are as follows. Although CPC has new implications in terms of the origin of human symbolic communication, including language, the CPC hypothesis does not explain why symbolic communication emerged only in humans and not in other living species. However, certain types of symbolic communication have also been observed in other living species (Rendall et al., 2009). The symbol emergence described in this paper is not argued to be strictly limited to humans. Considering that language and symbolic communication are multi-faceted phenomena, some types of the CPC may be found in other living species.

The CPC hypothesis focuses primarily on the semantic aspects of the SESs. Language, the most popular symbolic human system, is multi-faceted. Furthermore, the emergence of speech codes, such as phonological systems, is an important topic in the study of SESs. This study focuses on the emergence of the semantic aspects of symbol systems. However, the emergence of phonological systems is not discussed, although word discovery is mentioned in relation to speech signals in Section 3.2, from the viewpoint of PC by a single agent. Computational models for the self-organization of speech codes in multi-agent systems have also been studied for more than a decade (Oudeyer, 2005). In particular, the work by Moulin-Frier et al. (2015) proposed a Bayesian framework for speech communication and the emergence of a phonological system, termed COSMO (Communicating about Objects using Sensory–Motor Operations). Integrating this concept into the CPC framework may provide a possible path for creating a more general computational model for SESs. We believe that the CPC framework possesses the generality to accommodate such discussions.

Therefore, testing the CPC hypothesis is important. Tests may involve at least two computational and cognitive approaches. Computational models can be developed to enable AIs and robots to perform symbol emergence in a variety of tasks to test the feasibility of the CPC hypothesis in a constructive manner. Psychological experiments can also be conducted to determine whether humans actually perform the learning processes assumed in the CPC hypothesis. Particularly, Hagiwara et al. (2019) assumed that agents decide whether to accept or reject another agent’s utterance using a certain probability calculated based on their individual beliefs. The extent to which individuals act according to these assumptions must be validated. Okumura et al. (2023) conducted initial studies on the aforementioned topic and reported that human participants adhered to the acceptance probability suggested by the theory of the MH naming game to a certain extent. In addition, the extent to which the free energy of $w_{d}$ in Figure 7 can be minimized must be tested.

Understanding the dynamics of SESs that realize daily semiotic communications will contribute to understanding the origins of semiotic and linguistic communications. To enable robots to participate in daily human communication in the long term, the fundamental capability that enables humans to organize emergent symbol systems in a decentralized manner without a designer or centralized mechanism to create a language should be clarified. The CPC hypothesis, including the computational approach that decomposes the CPC into a decentralized individual representation of learning and communication, can be adapted to provide a general and promising direction for illuminating the mystery of the emergence of symbolic communications and language.

## Data Availability

The original contributions presented in the study are included in the article/Supplementary Material, further inquiries can be directed to the corresponding author.

## Footnotes

1 In this paper, we consider both “symbolic communication” and “semiotic communication” depending on the context and their relationship with relevant discussions and research. However, both can be considered to have the same meaning.

2
Ueda and Taniguchi (2024) provides the theory that connects these two approaches.

3 For example, when a certain amount of force is applied to an object (such as a brick wall), a corresponding force is returned as a reaction, hence when we strike an object with a certain force, we experience a certain level of response in terms of force. When we look at an apple, our visual system receives a sensation of the color red. These factors do not change significantly with the community to which the agent belongs. Here, “red” is distinguished as a sign and sensor signal, and its meaning is dependent on language, as the famous example of colors of the rainbow suggests. Thus, the physical information obtained by our visual system is not different. However, the perception, (i.e., categorization) is affected by linguistic or semiotic systems (Gliozzi et al., 2009; Deutscher, 2010; Althaus and Westermann, 2016). Although the meaning of a linguistic sign cannot be learned without semiotic communication with others, the physical properties of the sign can be learned from physical interactions with the target object without supervision because the physical properties of the object can be ascertained from the interaction.

4 Note that the idea of emergent properties here is different from that often mentioned recently in the context of foundation models, including LLMs (Bommasani et al., 2021).

5 The term “internal representations” is used in a more general sense than in representational theories of the mind (i.e., representationalism) in the theory of SESs. The internal representations in this study do not strictly refer to (physical) symbol systems that are often considered in classical representational accounts of the mind and AI. For example, the activation patterns in neural networks are regarded as a type of internal representation. Conversely, the internal representation system is not an amodal “symbol system.” Unlike the amodal physical symbol system in conventional symbolic AI, the representation system can be formed without symbolic information or language and does not require symbolism in the sense of logic and physical symbol systems (Newell, 1980). Such a system is similar to the perceptual symbol system in cognitive psychology proposed by Barsalou (1999). The perceptual symbol system theory (Barsalou, 1999) argues that bottom-up patterns of sensory-motor multi-modal data are associated with the perceptual process and that perceptual symbols are formed.

6 However, merely forming an internal representation system, which involves representation learning or categorization, is not sufficient for semiotic communication. This is because it is crucial to consider the degree of freedom associated with signs.

7 This is closely related to the discussion on the theory of linguistic relativity (i.e., Sapir–Whorf hypothesis)Deutscher (2010).

8 Essential software for controlling robots and their behaviors to obtain sensor information are programmed by human developers.

9
Taniguchi et al. (2017a) proposed spatial concept acquisition and simultaneous localization and mapping (SpCoSLAM) for spatial concept formation. SpCoSLAM integrated visual, positional, and (auditory) linguistic information to form a map, located the position of the robot, identified clusters of positions, and discovered words in a bottom-up manner. Although the detailed features of PGM differed from that of MLDA, SpCoSLAM could be regarded as a variant of a multi-modal categorization model. Additionally, SpCoSLAM was trained to predict observations and infer latent variables (i.e., spatial concepts) via Bayesian inference. These studies were related to semantic map formation in robotics (Kostavelis and Gasteratos, 2015; Garg et al., 2020).

10 The model described in this section is conceptual in the sense that the type of probabilistic distributions, precise architectures of the generative model, or inference procedures are not specified. By specifying these, a variety of concrete models of CPC (i.e., symbol emergence) can be obtained.
